# Retrospective childhood attention-deficit/hyperactivity disorder traits and their differential associations with mood disorder presentations in adulthood

**DOI:** 10.1007/s00406-026-02251-9

**Published:** 2026-04-24

**Authors:** Yewon Jwa, Jakyung Lee, Hyeona Yu, Chan Woo Lee, Hyun Jung Hur, Hyun Seok Do, Jongwook Lim, Shinn Won Lim, Jun Hyuck Kim, In-Ae Hwang, Hyo Shin Kang, Tae Hyon Ha, Jungkyu Park, Woojae Myung

**Affiliations:** 1 College of Medicine, Seoul National University, Seoul, Republic of Korea; 2 Department of Neuropsychiatry, Seoul National University Bundang Hospital, Seongnam, Republic of Korea; 3 Department of Psychology, Kyungpook National University, Daegu, Republic of Korea; 4 Department of Psychiatry, Seoul National University College of Medicine, Seoul, Republic of Korea; 5 Department of Health Science and Technology, Seoul National University, Seoul, Republic of Korea

**Keywords:** Childhood ADHD traits, Mood disorders, Major depressive disorder, Bipolar disorders, Clinical correlates, Emotion dysregulation

## Abstract

**Background:**

Childhood symptoms of Attention-Deficit/Hyperactivity Disorder (ADHD) often persist in adulthood and overlap considerably with mood disorder presentations. This study examines how specific childhood ADHD traits relate to distinct clinical characteristics in adults with mood disorders.

**Methods:**

We analyzed data from 755 adults diagnosed with major depressive disorder (*n* = 287) or bipolar disorder (*n* = 468). Participants retrospectively reported childhood ADHD traits using the 25-item Wender Utah Rating Scale. We mapped associations between childhood ADHD total score and three childhood ADHD traits (Impulsivity/Behavioral Problems, Inattentiveness/School Problems, Self-esteem/Negative Mood) and a comprehensive set of clinical variables, including depression, anxiety, affective temperament, emotional instability, adult ADHD symptoms, emotion regulation skills, childhood trauma, interpersonal sensitivity, resilience, problematic alcohol use. Partial correlations were used to identify initial links, followed by the path analysis to further explore these associations.

**Results:**

Childhood ADHD traits showed its strongest associations with trait-based mood instability, childhood trauma exposure, adult ADHD symptoms, and interpersonal sensitivity. Among mood temperaments, depressive temperament showed the strongest association, while hyperthymic the weakest. Emotion regulation skills showed minimal association. No significant subgroup differences were found among diagnostic groups or sexes according to multigroup SEM. Dimensionally, Self-esteem/Negative Mood uniquely predicted greater depressive and anxiety symptoms, behavioral inhibition, higher interpersonal sensitivity, lower resilience, and problematic alcohol use. Inattentiveness/School Problems was the strongest and most consistent predictor of adult ADHD symptoms. Impulsivity/Behavioral Problems selectively related to irritable temperament, behavioral activation, and lower interpersonal sensitivity.

**Conclusions:**

Childhood ADHD traits show distinct, dimension-specific associations with adult mood disorder phenotypes. Viewing ADHD as distinct traits may guide more personalized treatment strategies.

**Supplementary Information:**

The online version contains supplementary material available at 10.1007/s00406-026-02251-9.

## Introduction

Attention Deficit/Hyperactivity Disorder (ADHD) is a neurodevelopmental condition that presents with persistent patterns of inattention, hyperactivity, and impulsivity, which significantly impair academic performance, social functioning, and daily life activities [1–3]. Although ADHD is primarily classified as a neurodevelopmental disorder, ADHD traits often co-occur with mood disorders in adult clinical settings. This co-occurrence presents substantial diagnostic challenges due to the symptomatic similarities in features such as inattention and impulsivity, which often complicates the formulation of effective treatment plans, for instance, the cautious use of stimulants such as methylphenidate in patients with bipolar disorder [4–7].

Furthermore, a growing body of longitudinal research also suggests that childhood ADHD symptoms are associated with a more complicated clinical course in mood disorders. For instance, prospective studies have found that individuals with comorbid ADHD and major depressive disorder exhibit a higher incidence of diagnostic conversion to bipolar disorder later in life [8]. Moreover, longitudinal data demonstrate that childhood ADHD features remain a significant predictor of depressive severity through emerging adulthood [9], while comorbid ADHD and mood disorder are associated with notably high rates of additional psychiatric comorbidities such as anxiety disorder [10, 11].

Despite the well-established association between ADHD-related features and poorer clinical outcomes in the mood disorder population, the specific ways through which early ADHD traits related to adult mood disorder phenotypes remain incompletely investigated. ADHD is a multifaceted construct that can be decomposed into distinct dimensions [12, 13], and its impact on adult functioning may be dimension-specific rather than uniform. In this context, research has consistently documented sex differences in the developmental expression and recognition of ADHD, as well as in patterns of psychiatric comorbidity [14, 15].

Adults with mood disorders recalling childhood ADHD traits retrospectively have demonstrated reasonable validity, often aligning with prospective clinical records, especially regarding earlier onset and recurrent mood episodes [16, 17]. Building on this established validity, multiple cross-cultural validation studies have identified a consistent three-factor structure in widely used retrospective measures, such as the Wender Utah Rating Scale (WURS-25) [18]. This structural evidence confirms that childhood ADHD traits encompass not only core neurodevelopmental symptoms such as inattention and impulsivity/hyperactivity, but also emotional distress including negative mood and low self-esteem, which is considered an independent factor-derived dimension [19–21]. Although these emotional traits are often considered secondary or overlapping features, early emotional disturbances may be quantifiable phenotypes that must be captured, particularly in clinical populations that experience enduring emotional disturbances later in life.

However, the dimension-specific impact of early ADHD traits and their invariance or specificity across diagnoses remains unexplored. For example, prior work has primarily emphasized ADHD–BD overlap with regard to shared mood temperament, whereas comparable evidence in MDD is relatively limited [22]. Additionally, if there are possible connections between childhood ADHD traits and various manifestations of mood disorder phenotypes, such as lower psychological resilience being linked to a lower level of self-esteem and a depressed mood retrospectively, these early traits could serve as meaningful markers closely related to the detailed clinical presentations of patients with mood disorders. Therefore, there is a clear need for an exploratory approach that systematically examines the complex interrelationships and structural patterns between dimensions of childhood ADHD and a wide range of adult mood disorder manifestations.

The present study intends to gauge possible connection by testing dimension-level associations between retrospectively reported childhood ADHD traits (WURS-25 derived sub-factors: Impulsivity/Behavioral Problems, Inattentiveness/School Problems, Self-esteem/Negative Mood [18]) and a broad set of clinical characteristics in a well-characterized mood disorder cohort in an exploratory manner. Building on this framework, the study aims to (1) determine whether the three childhood ADHD-related trait dimensions show distinct association profiles with adult clinical features using partial correlation analysis to isolate the pure and independent contribution of each dimension, and to identify any potential differences between diagnostic subgroups and sex; and (2) employ path analysis to evaluate the comprehensive structural pathways, focusing on the unique influences of each childhood ADHD dimension on adult phenotypes. Clarifying these profiles can refine etiological models and guide personalized targets for assessment and treatment planning.

## Materials and methods

### Participants

Initially, a total of 2,025 patients who visited the Mood Disorder Clinic at Seoul National University Bundang Hospital between 2019 and 2024 were screened. Following the initial screening, 1,642 patients were identified as meeting the diagnostic criteria for a mood disorder. Diagnosis of MDD, BD I, and BD II were confirmed by two board-certified psychiatrists (W.M. and T.H.H.), using the Mini-International Neuropsychiatric Interview (M.I.N.I) [23], a comprehensive review of electronic medical records, or adherence to the Diagnostic and Statistical Manual of Mental Disorders, Fifth Edition (DSM-5) [1] criteria. At this stage, participants were excluded if they had neurological or medical conditions that could account for their mood episodes, or if their diagnosis fell outside the scope of MDD, BD I, and BD II, such as cases where anxiety disorders were the primary diagnosis.

Among these patients, 33 individuals were excluded for being under the age of 19, as the WURS-25 and Adult ADHD Self-Report Scale (ASRS) are designed for adult populations, leaving 1,609 eligible adults. Of these, 811 patients who completed the WURS-25 without missing values were retained. Finally, 56 participants were excluded because of missing data on other clinical measures or demographic information. Consequently, a final sample of 755 adults was included in the analysis (*n* = 287 with MDD; *n* = 468 with BD [*n* = 123 with BD I and *n* = 345 with BD II]). Because the Mood Instability Questionnaire-Trait (MIQ-T) was added to the assessment battery later in the study period, data for this specific measure were available only for a subset of the final sample (*n* = 430 for the total sample; *n* = 120 for MDD; *n* = 310 for BD; *n* = 135 for males; *n* = 295 for females). The procedures for informed consent and the research protocol were reviewed and obtained from the Institutional Review Board of Seoul National University Bundang Hospital (approval no. B-2205-756-111; initial approval date: 2022-05-02).

### Measurements

#### Retrospective childhood attention-deficit/hyperactivity disorder traits

We assessed childhood ADHD traits using the Korean version [24] of the 25-item Wender Utah Rating Scale (WURS-25), a retrospective self-report tool that evaluates ADHD-related behaviors and associated difficulties during childhood originally developed by Ward et al. [18]. Prior factor analytic study [21, 25] identified a three-factor structure underlying the WURS-25 that differs from the traditional DSM-based ADHD symptom presentations. These dimensions of the WURS-25 include: (1) Impulsivity/Behavioral Problems, comprising 13 items that capture impulsivity-related externalizing behaviors (e.g., temper outbursts/tantrums and trouble with authorities); (2) Inattentiveness/School Problems, comprising seven items that reflect attentional difficulties and academic underperformance (e.g., concentration problems and poor grades); and (3) Self-esteem/Negative Mood, comprising five items that represent internalizing symptoms such as low self-worth and dysphoric mood (e.g., feeling sad, depressed, or unhappy). Participants rated each item on a five-point scale ranging from 0 (not at all) to 4 (very much), with higher total scores indicating greater severity of retrospectively reported childhood ADHD traits. In the present sample, the WURS-25 total score demonstrated excellent reliability (Cronbach’s alpha = 0.94), and the three subscales showed good to excellent internal consistency: Impulsivity/Behavioral Problems (α = 0.92), Inattentiveness/School Problems (α = 0.82), and Self-esteem/Negative Mood (α = 0.85).

#### Clinical features linked to adult mood disorders

Clinical variables were assessed using the following standardized instruments. Depression and anxiety were assessed with the Korean translated version of Zung Self-Rating Depression Scale (Zung SDS) [26] and the Korean version of the Beck Anxiety Inventory (BAI) [27]. Affective temperament was measured with the Korean short version of the Temperament Evaluation of Memphis, Pisa, Paris, and San Diego Auto-questionnaire (TEMPS-A-SV) [28]. Mood instability was evaluated with the Mood Instability Questionnaire-Trait (MIQ-T) [29], an instrument originally developed and validated at the Seoul National University Bundang Hospital Mood Disorder Clinic to assess trait-like mood instability. Behavioral inhibition and activation were assessed with the Korean valiated version of the BIS/BAS scales [30]. Adult ADHD symptoms were measured with the Korean version of the Adult ADHD Self-Report Scale (ASRS) [31]. Adaptive emotion regulation skills were assessed with the Korean translation version of Emotion Regulation Skills Questionnaire (ERSQ) [32]. Resilience was measured with the Korean validated version of Connor-Davidson Resilience Scale (CD-RISC) [33]. Problematic alcohol use was measured with the Korean version of the Alcohol Use Disorders Identification Test (AUDIT) [34]. Childhood trauma exposure was measured with the Korean validated version of Childhood Trauma Questionnaire–Short Form (CTQ-SF) [35]. Interpersonal sensitivity was assessed with the Korean version of the Interpersonal Sensitivity Measure (IPSM) [36]. Circadian rhythm preferences were evaluated with the Korean validated version of Composite Scale of Morningness (CSM) [37] and seasonal effects on mood and behavior were assessed with the Korean translated version of Seasonal Pattern Assessment Questionnaire (SPAQ) [38]. The Supplementary Material provides detailed descriptions of each scale.

### Statistical analysis

#### Bivariate analysis

Partial correlations were calculated for the total sample (*N* = 755) to examine the associations between retrospectively reported childhood ADHD traits, which were measured by the WURS-25 total score and its three factor-derived subscales, and current clinical features. All analyses were adjusted for age, diagnosic status, sex, education level, employment status, marital status, alcohol use, and smoking. When AUDIT was the dependent variable, we excluded alcohol use from the covariate set. Subgroup analyses were repeated by diagnosis (MDD, *n* = 287; BD, *n* = 468) and sex (male, *n* = 245; female, *n* = 510). For variables available only in specific subsets (i.e., MIQ-T), we restricted the analyses to participants with complete data (*n* = 430 for the total sample; *n* = 120 for MDD; *n* = 310 for BD; *n* = 135 for males; *n* = 295 for females). Bonferroni correction was applied to all correlation tests to control the family-wise (Type I) error arising from multiple comparisons, with significance thresholds adjusted accordingly.

#### Multivariate path analysis

Path analysis was performed within a structural equation modeling (SEM) framework to identify the structural relationship between childhood ADHD traits and various psychiatric outcomes. In the path analysis model, the three WURS-25 subscales were entered as the exogenous predictors (independent variables), and the 11 psychiatric outcomes were entered as the endogenous variables (dependent variables). Contrary to the partial correlation analysis, TEMPS-A-SV, MIQ-T, and CTQ-SF were excluded from the analysis due to ambiguous directionality.

Before conducting the main path analysis, multi-group SEM (MGSEM) was employed to evaluate whether the structural relationship between three childhood ADHD traits and psychiatric outcomes were invariant across diagnosis groups (MDD vs. BD) and sex. To do so, we compared a free estimated model (with path coefficients allowed to vary across groups) to a full constrained model (with paths coefficients constrained to be equal) using the likelihood ratio test (LRT).

Although our constructs met the conventional normality criteria (Table S1) (skewness < ± 3; kurtosis < ± 10) [39], we employed the Maximum Likelihood Robust (MLR) estimator to enhance the robustness of parameter estimates against potential violations of other distributional assumptions commonly encountered in multivariate data [40]. The following covariates were adjusted for: age, sex, diagnostic group, education, employment status, marital status, and smoking status. To account for the theoretical interrelatedness of the constructs, covariances among the three childhood ADHD dimensions were allowed, and residual covariances among all 11 dependent variables were freely estimated and incorporated into the path analysis. Moreover, to account for potential inflation of the Type I error rate and ensure the robustness of our findings, the Benjamini-Hochberg False Discovery Rate (FDR) corrections were applied to the 33 primary pathways (3 predictors × 11 outcomes) to minimize Type I errors. We performed all analyses using R software (ver. 4.0.5; R Foundation for Statistical Computing, Vienna, Austria).

**Table 1:** Demographic characteristics of participants (*N* = 755)

Characteristics	Mean ± SD, *n* (%)
Age (years)	34.36 ± 12.39
Age range (n)	
10s (19+)	35 (4.6)
20s	317 (42.1)
30s	155 (20.5)
40s	129 (17.1)
50s	100 (13.2)
60s	19 (2.5)
Gender (%)	
Male	245 (32.5)
Female	510 (67.5)
Education (%)	
High school or below	183 (24.2)
Others	572 (75.8)
Employment status (%)	
Unemployed	459 (60.8)
Employed	296 (39.2)
Marital status (%)	
Married	262 (34.7)
Other (single, divorced, or widowed)	493 (65.3)
Alcohol use status (%)	
Alcohol consumer	323 (42.8)
Never (or alcohol abstinent)	432 (57.2)
Smoking status (%)	
Current smoker	178 (23.6)
Non-smoker	577 (76.4)

## Results

### Sample

The sample included 755 adults diagnosed with mood disorders, with a mean age of 34.36 years (*SD* = 12.39). Females comprised 67.5% of the sample, and 75.8% had completed education beyond high school. Regarding employment, 60.8% were unemployed, and 65.3% were single, divorced, or widowed. Additionally, 42.8% reported alcohol use, and 23.6% were current smokers (Table 1).

### Associations between childhood ADHD traits and clinical features

#### Total mood disorder group

**Fig. 1:**
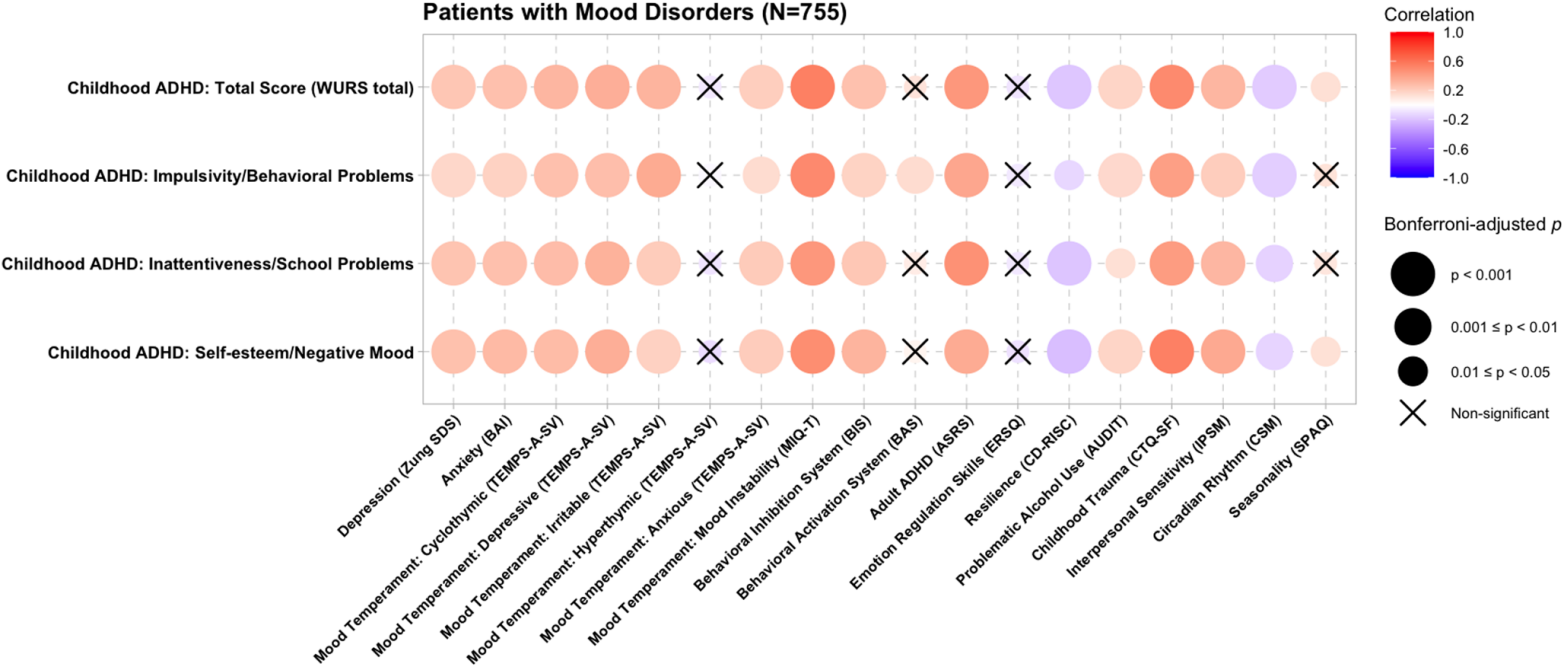
Correlation Matrix of Childhood ADHD, WURS-25 Derived Factor Dimensions, and Other Clinical Presentations (*N* = 755). *Note*. Partial correlations were adjusted for age, diagnosis, sex, education, employment, marital status, alcohol use status, and smoking status. For categorical variables, the reference categories (coded as 0) were: Male (Gender), High school or below (Education), Unemployed (Employment status), Other/Unmarried (Marital status), Alcohol abstinent (Alcohol use), and Non-smoker (Smoking status). For associations involving AUDIT, alcohol use status was not included as a covariate. X marks indicate non-significant associations (Bonferroni-corrected *p* > .05). Color intensity is proportional to the partial correlation coefficients. For the MIQ-T scale, the analysis was conducted in the subsamples that had the MIQ-T variable (*n* = 430). *Abbreviations* Zung SDS: Zung Self-Rating Depression Scale; BAI: Beck Anxiety Inventory; TEMPS-A-SV: Temperament Evaluation of Memphis, Pisa, Paris, and San Diego Autoquestionnaire-short version; MIQ-T: Mood Instability Questionnaire-Trait; BIS/BAS: The Behavior Inhibition System and Behavior Activation System scales; ASRS: Adult ADHD Self-Report Scale; ERSQ: Emotion Regulation Skills Questionnaire; CD-RISC: Connor–Davidson Resilience Scale; AUDIT: The Alcohol Use Disorder Identification Test-Korean version; CTQ-SF: Childhood Trauma Questionnaire-short form; IPSM: Interpersonal Sensitivity Measure; CSM: Composite Scale of Morningness; SPAQ: Seasonal Pattern Assessment Questionnaire

The partial correlation analysis conducted on the full sample (*N* = 755) revealed significant relationships between the WURS-25 total score, the three aspects of childhood ADHD traits, and various clinical measures (Fig. 1; Table 2). The current states of depression and anxiety (measured by Zung SDS and BAI, respectively) exhibited associations with the WURS-25 total score and all three traits. Self-esteem/Negative Mood demonstrated the strongest associations, with *r*_*p*_ = 0.26, *p* < .001 for depression and *r*_*p*_ = 0.29, *p* < .001 for anxiety.

When examining mood temperament using the TEMPS-A-SV, the WURS-25 total score showed moderate correlations with cyclothymic (*r*_*p*_ = 0.31 *p* < .001), depressive (*r*_*p*_ = 0.34, *p* < .001), and irritable (*r*_*p*_ = 0.32, *p* < .001) temperaments, and slightly weaker associations with anxious temperament (*r*_*p*_ = 0.21, *p* < .001). However, hyperthymic temperament showed negligible correlations (*r*_*p*_ = − 0.07, *p* = 1.000), and the three childhood ADHD traits showed a similar pattern.

Among the childhood ADHD traits, Self-esteem/Negative Mood showed the strongest association with depressive temperament (*r*_*p*_ = 0.34, *p* < .001), while Impulsivity/Behavioral Problems showed the highest correlation with irritable temperament (*r*_*p*_ = 0.36, *p* < .001). Trait-like mood instability, measured by the MIQ-T, exhibited strong correlations with the WURS-25 total score and all subdomain scores, with coefficients for Inattentiveness/School Problems (*r*_*p*_ = 0.45, *p* < .001), Self-esteem/Negative mood (*r*_*p*_ = 0.49, *p* < .001), and Impulsivity/Behavioral Problems (*r*_*p*_ = 0.50, *p* < .001).

Regarding the BIS/BAS, BIS showed stronger associations with childhood ADHD traits than BAS. BIS demonstrated a weak correlation with the WURS-25 total score (*r*_*p*_ = 0.26, *p* < .001), and its strongest subdomain correlation appeared for Self-esteem/Negative Mood (*r*_*p*_ = 0.32, *p* < .001). In contrast, BAS showed non-significant correlations childhood ADHD traits (*r*_*p*_ = 0.12, *p* = .111 for total score), except for a significant association with Impulsivity/Behavioral Problems (*r*_*p*_ = 0.15, *p* = .004).

Adult ADHD symptoms, assessed using the ASRS, showed a moderate correlation with the WURS-25 total score (*r*_*p*_ = 0.45, *p* < .001). Among the three traits, Inattentiveness/School Problems demonstrated the strongest association (*r*_*p*_ = 0.47, *p* < .001). We observed no significant associations between the WURS-25 total score or any of its childhood ADHD traits and current emotion regulation skills, as measured by the ERSQ (*p =* ns for all correlations). In contrast, we found a weak negative relationship between childhood ADHD traits and resilience (*r*_*p*_ = − 0.19, *p* < .001 for total score; *r*_*p*_ = − 0.19, *p* < .001 for Inattentiveness/School Problems; *r*_*p*_ = − 0.22, *p* < .001 for Self-esteem/Negative Mood), with the weakest association with Impulsivity/Behavioral Problems domain (*r*_*p*_ = − 0.13, *p* = .025). Furthermore, childhood ADHD traits were weak but significant associations with problematic alcohol use in adulthood (*r*_*p*_ = 0.18, *p* < .001 for total score).

Childhood ADHD traits also related to past and current problematic interpersonal experiences. Higher levels of childhood trauma exposure (CTQ-SF) showed moderate to strong correlations with childhood ADHD traits, with *r*_*p*_ = 0.50 (*p* < .001) for the WURS-25 total score and *r*_*p*_ = 0.54 (*p* < .001) for the Self-esteem/Negative Mood dimension. Current interpersonal sensitivity problems also showed moderate associations with *r*_*p*_ = 0.31 (*p* < .001) for the WURS-25 total score and *r*_*p*_ = 0.37 (*p* < .001) for Self-esteem/Negative Mood.

Chronotype, measured by the CSM, showed weak but significant negative associations with childhood ADHD traits. The WURS-25 total score correlated inversely with morningness–eveningness (*r*_*p*_ = − 0.17, *p* < .001), indicating that individuals with a stronger morningness preference reported fewer childhood ADHD traits. This weak inverse pattern appeared consistently across all childhood ADHD traits. Seasonal mood variability measured by the SPAQ, showed the weakest associations overall with childhood ADHD traits (*r*_*p*_ = 0.13, = 0.023, for total score).

#### Subgroup analyses

**Fig. 2:**
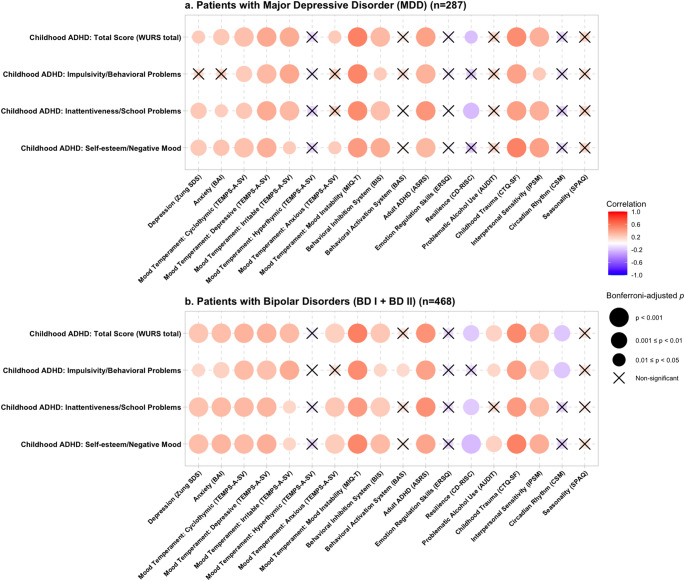
Correlation Matrix of Childhood ADHD, WURS-25 Derived Factor Dimensions, and Other Clinical Presentations in Patients with Major Depressive Disorder (*n* = 287) and Bipolar Disorders (*n* = 468**)**. *Note*. Partial correlations were adjusted for age, sex, education, employment, marital status, alcohol use status, and smoking status. For categorical variables, the reference categories (coded as 0) were: Male (Gender), High school or below (Education), Unemployed (Employment status), Other/Unmarried (Marital status), Alcohol abstinent (Alcohol use), and Non-smoker (Smoking status). For associations involving AUDIT, alcohol use status was not included as a covariate. X marks indicate non-significant associations (Bonferroni-corrected *p* > .05). Color intensity is proportional to the partial correlation coefficients. For the MIQ-T scale, the analysis was conducted in the subsamples that had the MIQ-T variable (*n* = 120 for MDD, *n* = 310 for BD). *Abbreviations* Zung SDS: Zung Self-Rating Depression Scale; BAI: Beck Anxiety Inventory; TEMPS-A-SV: Temperament Evaluation of Memphis, Pisa, Paris, and San Diego Autoquestionnaire-short version; MIQ-T: Mood Instability Questionnaire-Trait; BIS/BAS: The Behavior Inhibition System and Behavior Activation System scales; ASRS: Adult ADHD Self-Report Scale; ERSQ: Emotion Regulation Skills Questionnaire; CD-RISC: Connor–Davidson Resilience Scale; AUDIT: The Alcohol Use Disorder Identification Test-Korean version; CTQ-SF: Childhood Trauma Questionnaire-short form; IPSM: Interpersonal Sensitivity Measure; CSM: Composite Scale of Morningness; SPAQ: Seasonal Pattern Assessment Questionnaire

**Fig. 3:**
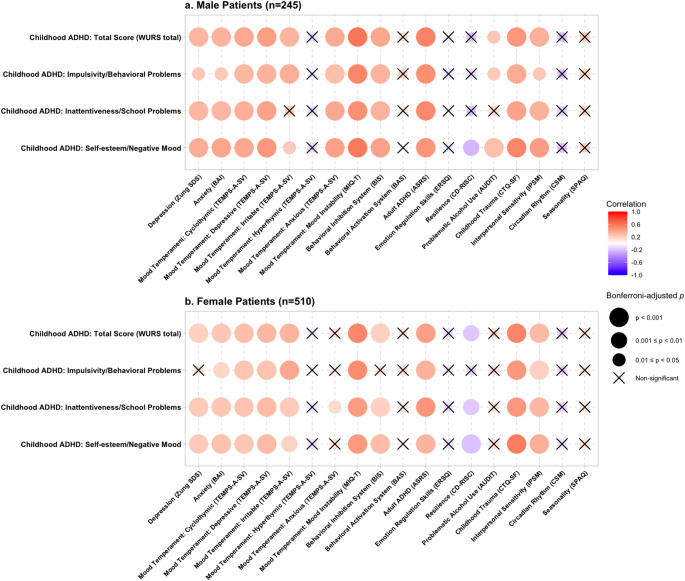
Correlation Matrix of Childhood ADHD, WURS-25 Derived Factor Dimensions, and Other Clinical Presentations in Male (*n* = 245) and Female Patients with Mood Disorder (*n* = 510)*Note*. Partial correlations were adjusted for age, diagnosis, education, employment, marital status, alcohol use status, and smoking status. For categorical variables, the reference categories (coded as 0) were: Male (Gender), High school or below (Education), Unemployed (Employment status), Other/Unmarried (Marital status), Alcohol abstinent (Alcohol use), and Non-smoker (Smoking status). For associations involving AUDIT, alcohol use status was not included as a covariate. X marks indicate non-significant associations (Bonferroni-corrected *p* > .05). Color intensity is proportional to the partial correlation coefficients. For the MIQ-T scale, the analysis was conducted in the subsamples that had the MIQ-T variable (*n* = 135 for Male, *n* = 295 for female). *Abbreviations* Zung SDS: Zung Self-Rating Depression Scale; BAI: Beck Anxiety Inventory; TEMPS-A-SV: Temperament Evaluation of Memphis, Pisa, Paris, and San Diego-Autoquestionnaire-short version; MIQ-T: Mood Instability Questionnaire-Trait; BIS/BAS: The Behavior Inhibition System and Behavior Activation System scales; ASRS: Adult ADHD Self-Report Scale; ERSQ: Emotion Regulation Skills Questionnaire; CD-RISC: Connor–Davidson Resilience Scale; AUDIT: The Alcohol Use Disorder Identification Test-Korean version; CTQ-SF: Childhood Trauma Questionnaire-short form; IPSM: Interpersonal Sensitivity Measure; CSM: Composite Scale of Morningness; SPAQ: Seasonal Pattern Assessment Questionnaire

Subgroup analyses revealed results that generally aligned with, yet also differed from, those in the overall sample (Figs. 2 and 3; Tables S2–S5). The association between Impulsivity/Behavioral Problems and current depression or anxiety, which reached significance in the total sample (Table 2), did not appear in patients with MDD (*r*_*p*_ = 0.16, *p* = .533 for depression; *r*_*p*_ = 0.19, *p* = .118 for anxiety). In the analysis of BD subgroup, a small but significant association persisted (*r*_*p*_ = 0.17, *p* = .014 for depression; *r*_*p*_ = 0.19, *p* = .002 for anxiety). In the analysis of male subgroup, Impulsivity/Behavioral Problems correlated with both depression and anxiety (*r*_*p*_ = 0.23, *p* = .020 for depression; *r*_*p*_ = 0.23, *p* = .020 for anxiety). In the analysis of female subgroup, the association was detected for anxiety but not for depression (*r*_*p*_ = 0.13, *p* = .191 for depression; *r*_*p*_ = 0.17, *p* = .008 for anxiety).

**Table 2:** Partial Correlation Between Childhood ADHD with WURS-25 Derived Factor Dimensions and Other Clinical Presentations (*N = 755*)

Variable	Zung SDS	BAI	TEMPS-A-SV (mood temperament)					MIQ-T^1^	BIS	BAS	ASRS	ERSQ	CD-RISC	AUDIT	CTQ-SF	IPSM	CSM	SPAQ
			Cyclothymic	Depressive	Irritable	Hyperthymic	Anxious											
Childhood ADHD: Total score	0.24 (< 0.001)	0.26 (< 0.001)	0.31 (< 0.001)	0.34 (< 0.001)	0.32 (< 0.001)	−0.07 (1.000)	0.21 (< 0.001)	0.54 (< 0.001)	0.26 (< 0.001)	0.12 (0.111)	0.45 (< 0.001)	−0.09 (1.000)	−0.19 (< 0.001)	0.18 (< 0.001)	0.50 (< 0.001)	0.31 (< 0.001)	−0.17 (< 0.001)	0.13 (0.023)
Impulsivity/ Behavioral Problems	0.17 (< 0.001)	0.19 (< 0.001)	0.26 (< 0.001)	0.27 (< 0.001)	0.36 (< 0.001)	−0.02 (1.000)	0.15 (0.004)	0.50 (< 0.001)	0.19 (< 0.001)	0.15 (0.004)	0.38 (< 0.001)	−0.07 (1.000)	−0.13 (0.025)	0.16 (< 0.001)	0.41 (< 0.001)	0.21 (< 0.001)	−0.16 (< 0.001)	0.12 (0.090)
Inattentiveness/ School Problems	0.25 (< 0.001)	0.27 (< 0.001)	0.28 (< 0.001)	0.33 (< 0.001)	0.22 (< 0.001)	−0.09 (1.000)	0.22 (< 0.001)	0.45 (< 0.001)	0.24 (< 0.001)	0.08 (1.000)	0.47 (< 0.001)	−0.08 (1.000)	−0.19 (< 0.001)	0.13 (0.021)	0.43 (< 0.001)	0.31 (< 0.001)	−0.15 (0.004)	0.10 (0.285)
Self-esteem/ Negative Mood	0.26 (< 0.001)	0.29 (< 0.001)	0.28 (< 0.001)	0.34 (< 0.001)	0.19 (< 0.001)	−0.11 (0.140)	0.21 (< 0.001)	0.49 (< 0.001)	0.32 (< 0.001)	0.04 (1.000)	0.36 (< 0.001)	−0.10 (0.537)	−0.22 (< 0.001)	0.18 (< 0.001)	0.54 (< 0.001)	0.37 (< 0.001)	−0.14 (0.006)	0.13 (0.032)

^1^The analysis was performed on a subsample (*n* = 430) that included the MIQ-T variable.

Note: Partial correlations were adjusted for age, diagnosis, sex, education, employment, marital status, alcohol use status, and smoking status. For categorical variables, the reference categories (coded as 0) were: Male (Gender), High school or below (Education), Unemployed (Employment status), Other/Unmarried (Marital status), Alcohol abstinent (Alcohol use), and Non-smoker (Smoking status). For associations involving AUDIT, alcohol use status was not included as a covariate; Three WURS-25 derived childhood ADHD traits were presented by Impulsivity/Behavioral Problems, Inattentiveness/School Problems, and Self-Esteem/Negative Mood; Exact Bonferroni-adujsted *p* values are reported to three decimals when *p* ≥ .001; adjusted *p* values greater than 1 were truncated to 1.000, reporting in parenthesis.

*Abbreviations* Zung SDS: Zung Self-Rating Depression Scale; BAI: Beck Anxiety Inventory; TEMPS-A-SV: Temperament Evaluation of Memphis, Pisa, Paris, and San Diego Autoquestionnaire-short version; MIQ-T: Mood Instability Questionnaire-Trait; BIS/BAS: The Behavior Inhibition System and Behavior Activation System scales; ASRS: Adult ADHD Self-Report Scale; ERSQ: Emotion Regulation Skills Questionnaire; CD-RISC: Connor–Davidson Resilience Scale; AUDIT: The Alcohol Use Disorder Identification Test-Korean version; CTQ-SF: Childhood Trauma Questionnaire-short form; IPSM: Interpersonal Sensitivity Measure; CSM: Composite Scale of Morningness; SPAQ: Seasonal Pattern Assessment Questionnaire

The partial correlation analysis identified varying patterns of associations in our subgroup analysis. Regarding temperament, significant correlations between childhood ADHD traits and cyclothymic, depressive, and anxious temperaments were observed in males (Table S4). In the anaylsis of female participants, the association with anxious temperament did not reach the significance (*r*_*p*_ = 0.12, *p* = .364) (Table S5). Notably, BAS showed a significant correlation with Impulsivity/Behavioral Problems within the BD subgroup (*r*_*p*_ = 0.16, *p* = .049). Among MDD participants, the Self-esteem/Negative Mood trait showed no significant association with resilience (*r*_*p*_ = − 0.20, *p* = .059) (Table S2).

In the domain of problematic alcohol use (AUDIT), significant correlations were observed in the BD subgroup and in males particularly in relation to Self-esteem/Negative Mood (*r*_*p*_ = 0.19, *p* = .002; *r*_*p*_ = 0.27, *p* < .001, respectively). However, no significant correlations were detected in the MDD subgroup or in females (*r*_*p*_ = 0.16, *p =* .540; *r*_*p*_ = 0.13, *p =* .226, respectively). Additionally, chronotype (CSM) showed significant negative correlations (greater eveningness) with Impulsivity/Behavioral Problems in BD (*r*_*p*_ = − 0.19, *p* = .003).

Several findings remained consistent across all subgroups. The general pattern of associations between childhood ADHD dimensions and depressive, cyclothymic, and irritable temperaments persisted (Tables S2–S5). Hyperthymic temperament showed no significant correlations with any childhood ADHD traits in any group. Trait-based mood instability (MIQ-T) demonstrated strong correlations across diagnostic and sex subgroups. Emotion regulation skills (ERSQ) did not relate to childhood ADHD traits across all subgroups. Lastly, among the three childhood ADHD traits, Impulsivity/Behavioral Problems generally exhibited relatively lower correlation coefficients with clinical variables compared to other childhood ADHD traits (Figs. 2 and 3; Tables S2–S5).

### Path analysis of childhood ADHD traits and current clinical features

**Fig. 4:**
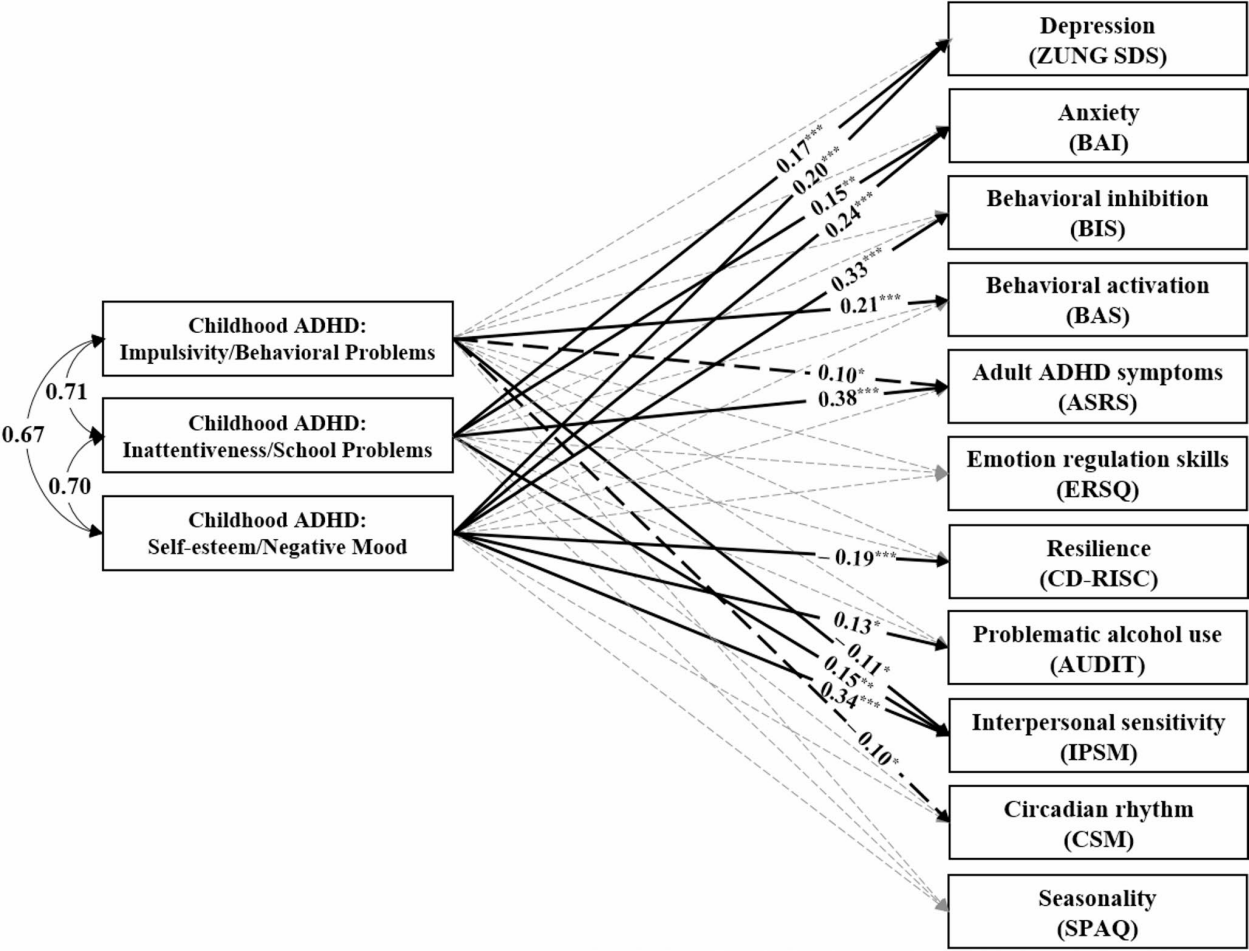
Pooled path model liking retrospective childhood ADHD traits to current clinical outcomes in patients with mood disorders. *Note.* Standardized coefficients are reported. Significant paths are indicated by bold solid lines, while non-significant paths are represented by dotted grey lines. The dotted bold black lines (Impulsivity/Behavioral Problems → Adult ADHD symptoms; Self-esteem/Negative mood → Circadian rhythms) indicate paths that were initially significant but became non-significant after applying False Discovery Rate (FDR) correction for multiple comparisons (3 independent variables × 11 psychiatric outcomes = 33 pairs). The model was adjusted for age, sex, diagnostic group, education, employment status, marital status, alcohol use, and smoking status; for the AUDIT model, alcohol use was excluded from the covariates to avoid conceptual redundancy. For categorical variables, the reference categories (coded as 0) were: Male (Gender), High school or below (Education), Unemployed (Employment status), Other/Unmarried (Marital status), Alcohol abstinent (Alcohol use), and Non-smoker (Smoking status). Covariances among the three childhood ADHD traits are visualized; residual covariances among all dependent variables were estimated in the model but are omitted from the figure for visual clarity. ^*^p<.05. ^**^p<.01. ^***^p < .001

The MGSEM revealed no significant differences between the two diagnostic groups and sex, suggesting structural invariance across these subgroups. For the diagnostic groups, the LRT showed no significant difference between the free and constrained models $\:(\varDelta\:{x}^{2}\left(33\right)=\mathrm{29,75},\:p=0.630).\:$Furthermore, the constrained model demonstrated a better fit based on information criteria (AIC = 53,774 vs. 53,808; BIC = 55,342 vs. 55,529). For sex differences, the overall LRT did not reach statistical significance$\:\:(\varDelta\:{x}^{2}\left(33\right)=45.43,\:p=0.073$), and the constrained model yielded lower AIC (53,766 vs. 53,784) and BIC (55,334 vs. 55,506) values. Given the evidence of structural invariance across groups, we utilized a unified pooled sample for the final path analysis, adjusting for age, diagnosis, sex, and other demographic variables as covariates (Fig. 4). All analyzed pathways are presented in Table 3, while the significant paths are specifically summarized and illustrated in Fig. 4.

Significant paths in the pooled path analysis among individuals with mood disorders are detailed in Table 3. While the path analysis already accounts for correlations between all variables in nature, additional False Discovery Rate (FDR) correction procedures were implemented to ensure robustness.

**Table 3:** Path analysis results (*N* = 755)

Dependent Variables	Independent Variables		Unstandardized Path Coefficient (95% CI)	S.E.	Standardized Path Coefficient		*p*	FDR *p*
Zung SDS	Impulsivity/Behavioral Problems		−0.07 (− 0.16, 0.01)	0.04	−0.08		0.087	0.179
	Inattentiveness/School Problems		0.30 (0.13, 0.46)	0.09	0.17		0.001	0.003
	Self-esteem/Negative Mood		0.39 (0.20, 0.58)	0.10	0.20		< 0.001	< 0.001
BAI	Impulsivity/Behavioral Problems		−0.08 (− 0.19, 0.04)	0.06	−0.06		0.191	0.300
	Inattentiveness/School Problems		0.33 (0.10, 0.56)	0.12	0.15		0.005	0.015
	Self-esteem/Negative Mood		0.60 (0.35, 0.85)	0.13	0.24		< 0.001	< 0.001
BIS	Impulsivity/Behavioral Problems		−0.02 (− 0.05, 0.01)	0.02	−0.06		0.213	0.320
	Inattentiveness/School Problems		0.04 (− 0.03, 0.10)	0.03	0.06		0.249	0.343
	Self-esteem/Negative Mood		0.22 (0.16, 0.29)	0.03	0.33		< 0.001	< 0.001
BAS	Impulsivity/Behavioral Problems		0.13 (0.06, 0.20)	0.04	0.21		< 0.001	0.001
	Inattentiveness/School Problems		0.00 (− 0.13, 0.13)	0.07	0.00		0.964	0.964
	Self-esteem/Negative Mood		−0.11 (− 0.26, 0.03)	0.07	−0.09		0.119	0.216
ASRS	Impulsivity/Behavioral Problems		0.11 (0.01, 0.21)	0.05	0.10		0.035	0.089
	Inattentiveness/School Problems		0.77 (0.57, 0.97)	0.10	0.38		< 0.001	< 0.001
	Self-esteem/Negative Mood		0.07 (− 0.14, 0.27)	0.10	0.03		0.525	0.619
ERSQ	Impulsivity/Behavioral Problems		0.00 (− 0.01, 0.01)	0.00	−0.01		0.838	0.900
	Inattentiveness/School Problems		0.00 (− 0.02, 0.01)	0.01	−0.01		0.846	0.900
	Self-esteem/Negative Mood		−0.01 (− 0.03, 0.00)	0.01	−0.09		0.110	0.213
CD-RISC	Impulsivity/Behavioral Problems		0.12 (− 0.08, 0.32)	0.10	0.07		0.237	0.340
	Inattentiveness/School Problems		−0.35 (− 0.71, 0.02)	0.19	−0.11		0.065	0.143
	Self-esteem/Negative Mood		−0.68 (− 1.07, − 0.29)	0.20	−0.19		0.001	0.003
AUDIT	Impulsivity/Behavioral Problems		0.06 (− 0.02, 0.15)	0.04	0.08		0.136	0.224
	Inattentiveness/School Problems		−0.03 (− 0.17, 0.11)	0.07	−0.02		0.679	0.773
	Self-esteem/Negative Mood		0.21 (0.05, 0.38)	0.08	0.13		0.012	0.037
IPSM	Impulsivity/Behavioral Problems		−0.17 (− 0.31, − 0.03)	0.07	−0.11		0.016	0.045
	Inattentiveness/School Problems		0.41 (0.14, 0.69)	0.14	0.15		0.003	0.010
	Self-esteem/Negative Mood		1.05 (0.75, 1.35)	0.15	0.34		< 0.001	< 0.001
CSM	Impulsivity/Behavioral Problems		−0.07 (− 0.14, 0.00)	0.03	−0.10		0.047	0.111
	Inattentiveness/School Problems		−0.06 (− 0.20, 0.07)	0.07	−0.05		0.359	0.445
	Self-esteem/Negative Mood		−0.06 (− 0.20, 0.07)	0.07	−0.04		0.364	0.445
SPAQ	Impulsivity/Behavioral Problems		0.03 (− 0.03, 0.08)	0.03	0.06		0.325	0.428
	Inattentiveness/School Problems		0.00 (− 0.09, 0.10)	0.05	0.01		0.924	0.953
	Self-esteem/Negative Mood		0.08 (− 0.02, 0.19)	0.05	0.09	0.124		0.216

Note: Age, sex, education, employment, marital status, alcohol use status, and smoking status were adjusted; For categorical variables, the reference categories (coded as 0) were: Male (Gender), High school or below (Education), Unemployed (Employment status), Other/Unmarried (Marital status), Alcohol abstinent (Alcohol use), and Non-smoker (Smoking status). The FDR p-value represents the False Discovery Rate (FDR) correction applied to multiple comparisons (3 independent variables × 11 psychiatric outcomes = 33 pairs); CI=confidence interval, *S.E*=standard error

*Abbreviations* Zung SDS: Zung Self-Rating Depression Scale; BAI: Beck Anxiety Inventory; BIS/BAS: The Behavior Inhibition System and Behavior Activation System scales; ASRS: Adult ADHD Self-Report Scale; ERSQ: Emotion Regulation Skills Questionnaire; CD-RISC: Connor–Davidson Resilience Scale; AUDIT: The Alcohol Use Disorder Identification Test-Korean version; IPSM: Interpersonal Sensitivity Measure; CSM: Composite Scale of Morningness; SPAQ: Seasonal Pattern Assessment Questionnaire

The Impulsivity/Behavioral Problems trait demonstrated a relatively narrow range of significant predictions. Specifically, higher levels of childhood impulsivity and behavioral problems were significant predictors of increased BAS scores (standardized path coefficient (β) = 0.21, *p* < .001, FDR *p* = .001). It also significantly a predicted lower interpersonal sensitivity (β = −0.11, *p* = .016, FDR *p* = .045). While this trait initially associated with adult ADHD symptoms (β = 0.11, *p* = .035), this relationship became not significant after FDR corrrection (FDR *p* = .089). Similarly, the association with eveningness preference did not remain significant after FDR correction (β = −0.10, *p* = .047, FDR *p* = .111).

The Inattentiveness/School Problems trait was primarily associated with adult ADHD symptoms (ASRS; β = 0.38, *p* < .001, FDR *p* < .001). Furthermore, it significantly predicted increased severity of current depression (Zung SDS; β = 0.17, *p* = .001, FDR *p* = .003), anxiety (BAI; β = 0.15, *p* = .005, FDR *p* = .015) and interpersonal sensitivity (IPSM; β = 0.15, *p* = .003, FDR *p* = .010).

The Self-esteem/Negative Mood traits emerged as the broadest predictor of adverse psychiatric outcomes in adulthood. Specifically, it predicted elevated depression (Zung SDS; β = 0.20, *p* < .001, FDR *p* < .001) and anxiety (BAI; β = 0.24, *p* < .001, FDR *p* < .001). This dimension was also positively linked to the Behavioral Inhibition System (BIS; β = 0.33, *p* < .001, FDR *p* < .001), resilience (CD-RISC; β = −0.19, *p* = .001, FDR *p* = .003) interpersonal sensitivity (IPSM; β = 0.34, *p* < .001, FDR *p* < .001), and problematic alcohol use (AUDIT; β = 0.13, *p* = .012, FDR *p* = .037). However, this dimension was not associated with adult ADHD symptoms as measured by the ASRS (β = 0.07, *p* = .525, FDR *p* = .619).

## Discussion

This study examined retrospectively reported childhood ADHD traits in adults with mood disorders and found clear, trait-specific profiles: Self-esteem/Negative Mood was linked to internalizing symptoms (depression/anxiety), depressive temperament, childhood trauma, interpersonal sensitivity, and lower resilience. Path analysis revealed that the trait was significantly associated with problematic alcohol use; however, this relationship was pronounced and specifically relevant within the BD and male subgroups, as further supported by partial correlation analysis. Inattentiveness/School Problems served as the only robust independent predictor of adult ADHD symptoms; this trait also relates to internalizing symptoms and interpersonal sensitivity. Impulsivity/Behavioral Problems related more selectively to behavioral activation system and to eveningness in BD only. Interestingly, while partial correlations might suggest otherwise, the pooled path analysis revealed a significant negative association between Impulsivity/Behavioral Problems and interpersonal sensitivity. Lastly, no childhood ADHD traits independently predicted emotion regulation skills. Furthermore, according to path analysis results, seasonality and circadian rhythms were only minimally associated with childhood ADHD traits.

Both Inattentiveness/School Problems and Self-esteem/Negative Mood traits maintained their unique effects on current levels of depression and anxiety, whereas Impulsivity/Behavioral Problems traits showed the weakest and non-significant associations particularly in MDD subsample. Consistent with prior research [17, 41], affective childhood ADHD traits were closely aligned with current depression and anxiety. Furthermore, our findings provide a tentative clue that the inattentiveness observed during depressed states might, in part, share a long-standing cognitive vulnerability with childhood ADHD traits. In contrast, Impulsivity/Behavioral Problems domain showed the weakest associations, with no significant associations in MDD subsample. Although adults with mood disorders frequently exhibit impulsivity and mood lability [42, 43], the findings suggest that such symptoms may not stem directly from childhood ADHD-related impulsivity.

In examining childhood ADHD traits and affective temperaments assessed by TEMPS-A-SV, we observed significant correlations between the WURS-25 total score and depressive, irritable, and cyclothymic temperaments, with a weaker yet significant association with anxious temperament. These results align with previous research suggesting that ADHD and mood disorders share affective temperamental vulnerabilities, particularly depressive, cyclothymic, and irritable traits, while hyperthymic temperament consistently shows no significant association [22]. Beyond ADHD–bipolar comorbidity, our findings show that these associations also characterize individuals with unipolar depression.

Childhood ADHD traits showed distinct associations with specific temperaments: Self-esteem/Negative Mood traits was most strongly related to cyclothymic temperament and depressive temperament. Inattentiveness/School Problems were also strongly associated with cyclothymic and depressive temperament. Impulsivity/Behavioral Problems domain was most robustly associated with irritable temperament. These patterns suggest that each childhood ADHD traits may be related to a distinct affective temperament. The patterns of associations were largely consistent across diagnostic subgroups, with hyperthymic temperament showing no significant associations, and anxious temperament more strongly associated with childhood ADHD traits in the BD group. Correlation analyses conducted within each sex subgroup revealed stronger associations between childhood ADHD traits and cyclothymic and depressive temperaments in males, whereas anxious temperament broadly correlated with childhood ADHD traits in males but only weakly linked to inattention in females. These findings highlight the importance of assessing childhood ADHD traits and considering how temperament profiles may manifest with varying patterns across sexes. Incorporating temperament profiles into clinical evaluations may support more individualized treatment planning for mood disorder patients who recall childhood ADHD traits.

The relationship between childhood ADHD traits, trait-based mood instability, and emotion regulation capacity reveals a critical distinction between enduring emotional dysregulation traits and acquired regulatory competencies. Accumulating evidence conceptualizes ADHD as a disorder of core emotional dysregulation [44], with affective lability, irritability, and low frustration tolerance as central features [45]. Similar dysregulation profiles appear across mood disorder diagnostic categories, and studies report heightened affective instability in BD and MDD [46]. Consistent with this literature, MIQ‑T, which measures emotional lability, directional mood shifts, seasonal patterns, and childhood instability, moderately correlated with total childhood ADHD symptoms and maintained nearly identical coefficients in MDD and BD subgroups.

MIQ‑T also showed moderate correlations with the Impulsivity/Behavioral Problems trait, highlighting the link between early impulsivity and persistent mood instability. In contrast, ERSQ, which measures the adaptive use of regulation, displayed only trivial correlations with any childhood ADHD traits, and none emerged as independent predictors in multivariable analyses of the pooled and all subgroups. This pattern suggests that present‑day regulation skills may remain relatively intact despite enduring dysregulation traits. Overall, the findings distinguish trait-level mood instability from later-developed regulatory skills and indicate that childhood ADHD traits do not necessarily associated with current deficits in emotion regulation.

The BIS/BAS findings revealed distinct profiles: Self-Esteem/Negative Mood trait was strongly associated with BIS across correlation and pooled path model, whereas Impulsivity/Behavior Problems trait selectively related to BAS. These results align with prior evidence linking heightened BIS sensitivity to internalizing symptoms and elevated BAS to impulsivity and externalizing behaviors [47, 48]. Extending this work, our results show that these BIS/BAS profiles remain relevant in adults with mood disorders and relate to retrospectively reported childhood ADHD traits.

Across the full sample and all subgroups, we observed clear association between childhood and adult ADHD symptoms particularly within Inattentiveness/School Problems trait. They showed the strongest positive relevance. Critically, this prominence remained consistent in the path analysis. These findings are consistent with prior literature suggesting that attentional difficulties often show a strong link with aduld ADHD manifestations [49, 50]. After adjusting for overlap among childhood ADHD traits, Self-Esteem/Negative Mood showed no independent association with adult ADHD severity. This suggests that reported childhood inattentiveness, rather than affective traits, may be the primarily correlate of adult ADHD symptoms, whereas affect childhood traits more closely relate to the internalizing burden [17, 41].

Retrospectively reported Self-Esteem/Negative Mood trait showed domain-specific associations with lower psychological resilience in adults with mood disorders. However, within the MDD subsample, Inattentiveness/School Problems was the only trait significantly linked to lower resilience. This specific finding in MDD may be partially understood by previous literature linking resilience deficits to cognitive vulnerabilities associated with executive or academic dysfunction [25]. In contrast, the remaining results align with models suggesting that affective and self-concept vulnerabilities may be related to less adaptive coping strategies [51].

Overall, our study found only a weak association between Self-Esteem/Negative Mood trait and problematic alcohol use. Partial correation shows this relationship is primarily observed within the BD and male subgroups, suggesting that the link between recalled early affective states and alcohol use in adulthood may be specific to certain clinical population. Future research should explore sex-specific patterns, divergent drinking motives, or clinical heterogeneity.

In the present sample, all childhood ADHD traits demonstrated moderate positive associations with childhood trauma exposure, with the Self-Esteem/Negative Mood trait demonstrated the most prominent association. Given that both childhood trauma and the presence of childhood ADHD are established risk factors for a more severe treatment course in mood disorders [6, 52], and considering our findings that these childhood ADHD traits are linked to various psychiatric outcomes, their co-occurence may contribute to the overall clinical burden observed in patients with mood disorders. Future studies may investigate the intricate interconnectedness of trauma exposure and childhood ADHD traits with longitudinal symptom trajectories and treatment responsiveness in this population.

While interpersonal sensitivity was positively correlated with all childhood ADHD traits in the correlation analysis, controlling for other ADHD traits revealed a significant negative association, despite the positive bivariate correlations. This sign reversal likely reflects clinical heterogeneity within the mood disorder population rather than suggesting that impulsivity inherently leads to lower social sensitivity. As previous literature suggests, distinct profiles, such as individuals with high social anxiety who may exhibit discrete levels of impulsivity, can coexist within clinical samples [53, 54]. Therefore, although impulsivity is disinhibitory at its core, its clinical manifestation is complex, influenced by interactions with other affective and cognitive vulnerabilities. This complexity is further supported considering the high intercorrelations among childhood ADHD traits observed in our sample.

The study found a nominally significant, weak association between childhood ADHD traits particularly in the Impulsivity/Behavioral domain and eveningness preference. Although this association did not survive multiple comparison correction, it was notably evident within the BD subgroup, while no such associations were observed in any other subgroups. Diverging from findings in non-clinical samples, where eveningness related more closely to inattention [55, 56], and considering the established circadian disruptions and eveningness in BD [57], this pattern may reflect a unique phenotype overlap between impulsivity and circadian disruptions specifically in bipolar pathway [58]. However, given the small effect size, the retrospective nature of the data, and the absence of sex differences in our MGSEM, caution when interpreting the results. In contrast, seasonal variation in mood showed no significant associations with childhood ADHD traits in any analysis, suggesting minimal linkage between these constructs in the mood disorder population.

## Limitations and future research

Several limitations warrant consideration. First, the cross-sectional design precludes causal inferences because it captures retrospectively reported childhood ADHD traits and current mood-related features at a single time point. While our results suggest potential links, longitudinal studies are necessary to clarify the temporal and directional relationships between early ADHD traits and adult mood disorders phenotypes. Second, emotional dysregulation, commonly observed in ADHD and mood disorders, may confound efforts to differentiate the two conditions. The symptomatic overlap may complicate the interpretation of ADHD-specific effects. Third, we did not employ structured clinical interviews or formal diagnostic procedures to confirm ADHD diagnoses. As such, our findings pertain to ADHD-like traits reported within mood disorder populations, rather than to diagnostically verified ADHD cases. Most importantly, the use of the WURS-25 to assess childhood ADHD traits is inherently retrospective, introducing the possibility of recall bias. To mitigate this issue, we used path analysis to statistically control for the overlap in variances among the different constructs. Notably, the partial correlation between current depressive status and ADHD traits was weak (*r*_*p*_ = 0.26, *p* < .001), which supports the discriminant validity of these constructs. This finding suggests that they are not merely reflections of a single underlying factor. However, since patients with mood disorders may exhibit mood-congruent cognitive biases, wherein current emotional states influence recollection of early experiences, the potential for systematic reporting bias cannot be entirely ruled out. Additionally, the exclusive reliance on self-report instruments across all major variables may introduce shared method bias, which can artificially inflate observed correlations. Future studies should incorporate a broader range of informants, such as clinician ratings, to mitigate such biases and offer a more comprehensive understanding of symptom presentations. Moreover, the specific clinical and socio-demographic profile of our sample may limit the generalizability of the findings. The notably high unemployment rate (60.8%) and the lack of detailed data on medication use or current mood states (e.g., euthymic vs. acute episodes) at the time of assessment could act as confounding factors. Although we statistically controlled for employment status to mitigate its influence, the psychological stressors of unemployment and the potential impact of pharmacological treatments on symptom reporting remain unaddressed. Lastly, restricting our analysis solely to complete cases may have introduced selection bias, which could limit the representativeness and generalizability of our findings.

Despite these limitations, this study provides novel evidence that retrospectively reported childhood ADHD traits show trait-specific associations with key clinical constructs in adults with mood disorders. Specification of childhood ADHD traits may help identify at-risk individuals and deepen our understanding of psychopathological heterogeneity across mood disorders.

## Supplementary Information

Below is the link to the electronic supplementary material.

Supplementary file1

## Data Availability

Data are available upon request from the correspondence author, [WM].
