# Supplementary material for: Retrospective childhood attention-deficit/hyperactivity disorder traits and their differential associations with mood disorder presentations in adulthood
Source: Eur Arch Psychiatry Clin Neurosci. 2026 Apr 24;276(5):2425–40. doi: 10.1007/s00406-026-02251-9 (PMC13481475; doi:10.1007/s00406-026-02251-9)
Supplement: Supplementary file 1 [file 406_2026_2251_MOESM1_ESM.docx]

**Supplementary Materials**

**Retrospective Childhood Attention-Deficit/Hyperactivity Disorder Traits and Their Differential Associations with Mood Disorder Presentations in Adulthood**

**1. Supplementary Text**

*2.2. Measurements*

*2.2.2 Mood-related clinical features*

*Zung self-rating depression scale (Zung SDS)* We used Zung SDS to assess the severity of depression. Originally developed by [1], we employed the Korean translated version of the scale [2]. It consists of 20 items, with responses ranging from 1 (‘Not at all’) to 4 (‘Always’) on a four-point scale. This self-report questionnaire allows the patient to evaluate their current state of depressive symptoms, with higher scores reflecting more severe levels of depression. It has been shown to have good internal consistency in the Korean population (Cronbach’s alpha = 0.81) [3].

*The Beck Anxiety Inventory (BAI)* he Korean version of the Beck Anxiety Inventory (BAI) was utilized to assess the severity of anxiety [4]. The BAI consists of 21 items, with each item rated on a scale from 0 (not at all) to 3 (severely), based on the severity of symptoms experienced in the past week.The scale was proven reliable for both clinical and non-clinical Korean populations.

*Temperament Evaluation of Memphis, Pisa, Paris, and San Diego-Autoquestionnaire (TEMPSA-SV)* Mood temperament is assessed by the Korean validated short version of the Temperament Evaluation of Memphis, Pisa, Paris, and San Diego Autoquestionnaire (TEMPSA-SV), a 39-item self-report measure with a dichotomous response format (Yes = 1, No = 0) [5], derived from the original 110-item version rooted in the bipolar spectrum framework [6]. It assesses five major types of temperament (i.e., depressive, cyclothymic, hyperthymic, anxious, and irritable) and has proven to be a reliable tool for investigating the impact of temperament on mood disorders [6, 7].

*Mood Instability Questionnaire-Trait (MIQ-T)* Trait-based mood instability was assessed using the Mood Instability Questionnaire-Trait (MIQ-T), consisting of 59 items [8]. This scale was originally developed and validated at the Mood Disorders Clinic of Seoul National University Bundang Hospital to measure the temperamental aspects of mood instability in clinical populations with mood disorders. It evaluates five key dimensions of MI: Lability (e.g., “I constantly switch from being lively and sluggish.”), Downward Tendency (e.g., “When I experience something difficult, I get depressed easily.”), Upward Tendency (e.g., “When I see an opportunity for something I like, I get excited right away.”), Seasonality (e.g., “My vitality (physical and mental energy) changes depending on the season.”), and Childhood Instability (e.g., “As a child, I was hot- or short-tempered, with a low boiling point.”). A higher total score indicates that participants exhibit greater predisposed mood fluctuations. The MIQ-T has demonstrated high internal consistency and strong psychometric properties. Although it comprises several subscales, the total score was employed in this study, with higher scores reflecting greater predisposed, temperament-based mood fluctuations. The MIQ-T has been psychometrically validated, with its original developers demonstrating excellent internal consistency (α = 0.96 for the full scale).

*Behavioral Inhibition System and Behavioral Activation System scales (BIS/BAS)* The BIS/BAS Scales are self-report questionnaires developed to assess individual behavioral inhibition and activation systems, helping understand an individual’s motivation and reactivity [9]. We used the Korean version of the scale, which has demonstrated good psychometric properties in the Korean context [10]. BIS assesses risk avoidance and anxiety responses, while BAS evaluates reward seeking and positive reactions, subdivided into reward sensitivity, goal pursuit, and impulsivity. Patients responded on a Likert scale from 1 (“strongly disagree”) to 4 (“strongly agree”). Higher scores indicate greater sensitivity.

*Korean version of the adult ADHD self-report scale (K-ASRS)* The Korean version of ASRS was developed by the World Health Organization (WHO) to evaluate ADHD symptoms in adults [11]. This 18-item self-report questionnaire assesses issues related to inattention (e.g., “difficulty concentrating”) and hyperactivity/impulsivity (e.g., “fidgeting”) over the past six months. Responses are scored on a scale from 1 (“never”) to 5 (“very often”), with higher scores reflecting greater severity of ADHD symptoms.

*Emotion Regulation Skills Questionnaire (ERSQ)* Emotion Regulation Skills Questionnaire (ERSQ) is a 27-item self-report measure designed to assess a wide range of emotion regulation skills. Items are rated on a 5-point Likert scale 0 (“not at all”) to 4 (“almost always”) [12]. In the current study, we utilized the Korean version of the ERSQ provided by the original author [13]. The questionnaire includes nine theoretically derived subscales aimed at evaluating specific skills: awareness, sensation, clarity, understanding, acceptance, resilience, self-support, tolerance, and modification. While a formal Korean validation study is yet to be published, the psychometric validity of this translated version has been supported and employed in previous research (ω for the total composite = 0.97) [14].

*The Connor–Davidson Resilience Scale (CD-RISC)* The Connor–Davidson Resilience Scale (CD-RISC) is a self-report measure designed to assess an individual’s capacity to adapt to change and manage adversity [15]. This study utilized the Korean validated version of the CD-RISC [16], which comprises 25 items, each rated on a 5-point Likert scale ranging from 0 (“not true at all”) to 4 (“true nearly all the time”). The total score ranges from 0 to 100, with higher scores indicating greater resilience. The validity of Korean validated version has been supported (α = 0.95) [17].

*Interpersonal Sensitivity Measure (IPSM)* Patients’ overall sensitivity to interpersonal rejection was assessed by the Korean version of the Interpersonal sensitivity measure (IPSM-K) [18], , based on the original scale developed by Boyce and Parker [19]. It consists of five subscales: interpersonal awareness, need for approval, separation anxiety, shyness, and fragile inner self. However, the full scale was employed as a comprehensive indicator of rejection sensitivity. Participants provided responses on a 4-point Likert scale, ranging from 1 (“very unlike me”) to 4 (“very like me”). Higher scores on the Korean validated version of IPSM reflected a greater predisposition to sensitivity toward interpersonal rejection.

*Childhood Trauma Questionnaire-Short Form (CTQ-SF)* Childhood trauma was evaluated using the Childhood Trauma Questionnaire-Short Form (CTQ-SF), a retrospective self-report measure designed to capture the type and severity of trauma experienced before the age of 18 [20]. We employed the validated Korean version [16], encompassing five categories of trauma: emotional abuse, physical abuse, sexual abuse, emotional neglect, and physical neglect. The questionnaire originally comprises 28 items scored on a five-point Likert scale ranging from 1 (never true) to 5 (very often true). For this study, the three items related to minimization or denial were excluded, and the remaining 25 items were used to quantify the severity of childhood trauma. Higher total scores indicated greater exposure to childhood trauma.

*Alcohol Use Disorders Identification Test (AUDIT)* The AUDIT is a self-report questionnaire developed to detect and assess alcohol use disorders early [21]. For this study, the Korean validated version of the K-AUDIT was used to measure problematic alcohol usage [22]. It measures alcohol consumption patterns and related problems. Comprising 10 items, it evaluates alcohol consumption frequency, amount, and consequences. Each item is scored from 0 to 4, with a total score ranging from 0 to 40, classifying the risk level. It is simple and quick, widely used in clinical settings to assess alcohol use issues.

*The Composite Scale of Morningness (CSM)* The circadian rhythm was assessed using the CSM, a 13-item tool designed to evaluate circadian preference [23]. We used the Korean validated version of the CSM [24]. The CSM total score ranges from 13 to 55, with lower scores reflecting a preference for eveningness (“evening type”) and higher scores indicating a preference for morningness (“morning type”).

*The Seasonal Pattern Assessment Questionnaire (SPAQ)* Seasonal effects on mood and behavior were evaluated by SPAQ, which is a self-report instrument comprising 30 items that evaluate seasonal variations in mood and behavior [25]. We employed the Korean version [26]. The SPAQ includes the Global Seasonality Score (GSS), which quantifies the extent to which these changes affect daily life. Higher scores on the GSS were interpreted as reflecting a greater influence of seasonality on the individual.

**Table S1** Descriptive Statistics of Clinical Variables

| Variable | Mean | SD | Range | Min | Max | Skewness | Kurtosis |
| --- | --- | --- | --- | --- | --- | --- | --- |
| Childhood ADHD: Total | 26.75 | 20.68 | 100 | 0 | 100 | 0.88 | 0.24 |
| Childhood ADHD:  Impulsivity/Behavioral Problems | 12.24 | 11.35 | 52 | 0 | 52 | 1.09 | 0.45 |
| Childhood ADHD:  Inattentiveness/School Problems | 7.60 | 6.17 | 28 | 0 | 28 | 0.92 | 0.38 |
| Childhood ADHD:  Self-esteem/Negative Mood | 6.91 | 5.51 | 20 | 0 | 20 | 0.57 | -0.70 |
| ZUNG SDS | 53.18 | 10.78 | 56 | 23 | 79 | -0.48 | -0.35 |
| BAI | 21.83 | 13.93 | 59 | 0 | 59 | 0.45 | -0.62 |
| Mood temperament: cyclothymic | 6.49 | 3.62 | 12 | 0 | 12 | -0.21 | -1.14 |
| Mood temperament: depressive | 3.73 | 2.42 | 8 | 0 | 8 | 0.06 | -1.10 |
| Mood temperament: irritable | 2.20 | 2.10 | 8 | 0 | 8 | 0.78 | -0.33 |
| Mood temperament: hyperthymic | 2.74 | 2.20 | 8 | 0 | 8 | 0.48 | -0.74 |
| Mood temperament: anxious | 1.43 | 1.13 | 3 | 0 | 3 | 0.09 | -1.37 |
| MIQ-T | 97.87 | 28.77 | 142 | 26 | 168 | -0.24 | -0.62 |
| BIS | 22.65 | 3.79 | 18 | 10 | 28 | -0.64 | -0.17 |
| BAS | 35.80 | 7.20 | 38 | 14 | 52 | -0.18 | -0.23 |
| ASRS | 47.60 | 12.59 | 72 | 18 | 90 | 0.31 | 0.02 |
| ERSQ | 1.78 | 0.77 | 4 | 0 | 4 | 0.18 | -0.10 |
| CD-RISC | 45.42 | 19.56 | 98 | 2 | 100 | 0.12 | -0.48 |
| AUDIT | 7.21 | 8.69 | 37 | 0 | 37 | 1.39 | 1.14 |
| CTQ-SF | 52.05 | 18.65 | 99 | 25 | 124 | 0.83 | 0.32 |
| IPSM | 98.97 | 17.25 | 88 | 49 | 137 | -0.27 | -0.34 |
| CSM | 29.07 | 8.10 | 53 | 0 | 53 | 0.27 | -0.38 |
| SPAQ | 5.97 | 5.16 | 24 | 0 | 24 | 0.74 | -0.13 |

Abbreviations Zung SDS: Zung Self-Rating Depression Scale; BAI: Beck Anxiety Inventory; MIQ-T: Mood Instability Questionnaire-Trait; BIS/BAS: The Behavior Inhibition System and Behavior Activation System scales; ASRS: Adult ADHD Self-Report Scale; ERSQ: Emotion Regulation Skills Questionnaire; CD-RISC: Connor–Davidson Resilience Scale; AUDIT: The Alcohol Use Disorder Identification Test-Korean version; CTQ-SF: Childhood Trauma Questionnaire-short form; IPSM: Interpersonal Sensitivity Measure; CSM: Composite Scale of Morningness; SPAQ: Seasonal Pattern Assessment Questionnaire.

| Variable | **Zung SDS** | **BAI** | TEMPS-A-SV (mood temperament) | | | | | **MIQ-T^1^** | **BIS** | **BAS** | **ASRS** | **ERSQ** | **CD-RISC** | **AUDIT** | **CTQ** | **IPSM** | **CSM** | **SPAQ** |
| --- | --- | --- | --- | --- | --- | --- | --- | --- | --- | --- | --- | --- | --- | --- | --- | --- | --- | --- |
|  |  |  | **Cyclothymic** | **Depressive** | **Irritable** | **Hyperthymic** | **Anxious** |  |  |  |  |  |  |  |  |  |  |  |
| Childhood ADHD:  Total score | .22^*^ | .23^**^ | .27^***^ | .36^***^ | .35^***^ | -.11 | .22^*^ | .54^***^ | .30^***^ | .08 | .40^***^ | -.05 | -.21^*^ | .16 | .49^***^ | .34^***^ | -.14 | .16 |
| Impulsivity/  Behavioral Problems | .16 | .19 | .22^**^ | .30^***^ | .37^***^ | -.07 | .18 | .52^***^ | .22^*^ | .12 | .33^***^ | -.07 | -.16 | .15 | .40^***^ | .22^*^ | -.10 | .12 |
| Inattentiveness/  School Problems | .23^**^ | .21^*^ | .24^**^ | .36^***^ | .30^***^ | -.14 | .19 | .50^***^ | .27^***^ | .02 | .46^***^ | -.02 | -.23^**^ | .12 | .41^***^ | .34^***^ | -.16 | .17 |
| Self-esteem/  Negative Mood | .23^**^ | .25^**^ | .26^***^ | .34^***^ | .22^*^ | -.13 | .21^*^ | .42^***^ | .35^***^ | .03 | .30^***^ | -.03 | -.20 | .16 | .54^***^ | .40^***^ | -.12 | .16 |

**Table S2.** Correlation between Childhood ADHD with its Factor Dimensions and Other Clinical Presentations in Patients with **Major Depressive Disorder (*N* = 287)**

^1^The analysis was conducted in the subsamples (n=120) who had the MIQ-T variable;
Note: Age, sex, education, employment, marital status, alcohol use status, and smoking status were adjusted; Adjusted p-values with Bonferroni’s correction were presented ^*^*p*<.05, ^**^*p*<.01, ^***^*p*<.001;
The subdomains of childhood ADHD symptoms were presented by Impulsivity/Behavioral Problems, Inattentiveness/School Problems, and Self-Esteem/Negative Mood.

*Abbreviations* Zung SDS: Zung Self-Rating Depression Scale; BAI: Beck Anxiety Inventory; TEMPS-A-SV: Temperament Evaluation of Memphis, Pisa, Paris, and San Diego-Autoquestionnaire-short version; MIQ-T: Mood Instability Questionnaire-Trait; BIS/BAS: The Behavior Inhibition System and Behavior Activation System scales; ASRS: Adult ADHD Self-Report Scale; ERSQ: Emotion Regulation Skills Questionnaire; CD-RISC: Connor–Davidson Resilience Scale ; AUDIT: The Alcohol Use Disorder Identification Test-Korean version; CTQ: Childhood Trauma Questionnaire-short form; IPSM: Interpersonal Sensitivity Measure; CSM: Composite Scale of Morningness; SPAQ: Seasonal Pattern Assessment Questionnaire.

| Variable | **Zung SDS** | **BAI** | TEMPS-A-SV (mood temperament) | | | | | **MIQ-T^1^** | **BIS** | **BAS** | **ASRS** | **ERSQ** | **CD-RISC** | **AUDIT** | **CTQ** | **IPSM** | **CSM** | **SPAQ** |
| --- | --- | --- | --- | --- | --- | --- | --- | --- | --- | --- | --- | --- | --- | --- | --- | --- | --- | --- |
|  |  |  | **Cyclothymic** | **Depressive** | **Irritable** | **Hyperthymic** | **Anxious** |  |  |  |  |  |  |  |  |  |  |  |
| Childhood ADHD:  Total score | .25^***^ | .28^***^ | .32^***^ | .33^***^ | .29^***^ | -.05 | .20^***^ | .55^***^ | .24^***^ | .13 | .47^***^ | -.11 | -.18^**^ | .19^**^ | .51^***^ | .29^***^ | -.19^**^ | .11 |
| Impulsivity/   Behavioral Problems | .17 | .19^*^ | .28^***^ | .27^***^ | .36^***^ | .00 | .13 | .49^***^ | .17^*^ | .16^*^ | .40^***^ | -.08 | -.12 | .17^*^ | .42^***^ | .21^***^ | -.19^**^ | .11 |
| Inattentiveness/  School Problems | .26^***^ | .29^***^ | .30^***^ | .31^***^ | .17^*^ | -.06 | .24^***^ | .43^***^ | .22^***^ | .10 | .48^***^ | -.11 | -.18^**^ | .14 | .43^***^ | .29^***^ | -.14 | .07 |
| Self-esteem/  Negative Mood | .28^***^ | .31^***^ | .29^***^ | .34^***^ | .17^*^ | -.11 | .22^***^ | .51^***^ | .29^***^ | .05 | .39^***^ | -.13 | -.23^***^ | .19^**^ | .54^***^ | .35^***^ | -.15 | .11 |

**Table S3.** Correlation between Childhood ADHD with its Factor Dimensions and Other Clinical Presentations in Patients with **Bipolar Disorder (*N* = 468)**

^1^The analysis was conducted in the subsamples (n=310) who had the MIQ-T variable.
Note: Age, sex, education, employment, marital status, alcohol use status, and smoking status were adjusted; Adjusted p-values with Bonferroni’s correction were presented ^*^*p*<.05, ^**^*p*<.01, ^***^*p*<.001;
The subdomains of childhood ADHD symptoms were presented by Impulsivity/Behavioral Problems, Inattentiveness/School Problems, and Self-Esteem/Negative Mood.

*Abbreviations* Zung SDS: Zung Self-Rating Depression Scale; BAI: Beck Anxiety Inventory; TEMPS-A-SV: Temperament Evaluation of Memphis, Pisa, Paris, and San Diego-Autoquestionnaire-short version; MIQ-T: MIQ-T: Mood Instability Questionnaire-Trait; BIS/BAS: The Behavior Inhibition System and Behavior Activation System scales; ASRS: Adult ADHD Self-Report Scale; ERSQ: Emotion Regulation Skills Questionnaire; CD-RISC: Connor–Davidson Resilience Scale ; AUDIT: The Alcohol Use Disorder Identification Test-Korean version; CTQ: Childhood Trauma Questionnaire-short form; IPSM: Interpersonal Sensitivity Measure; CSM: Composite Scale of Morningness; SPAQ: Seasonal Pattern Assessment Questionnaire.

| Variable | **Zung SDS** | **BAI** | TEMPS-A-SV (mood temperament) | | | | | **MIQ-T^1^** | **BIS** | **BAS** | **ASRS** | **ERSQ** | **CD-RISC** | **AUDIT** | **CTQ** | **IPSM** | **CSM** | **SPAQ** |
| --- | --- | --- | --- | --- | --- | --- | --- | --- | --- | --- | --- | --- | --- | --- | --- | --- | --- | --- |
|  |  |  | **Cyclothymic** | **Depressive** | **Irritable** | **Hyperthymic** | **Anxious** |  |  |  |  |  |  |  |  |  |  |  |
| Childhood ADHD:  Total score | .31^***^ | .32^***^ | .37^***^ | .41^***^ | .31^***^ | -.10 | .36^***^ | .59^***^ | .37^***^ | .14 | .53^***^ | -.06 | -.19 | .24^*^ | .45^***^ | .33^***^ | -.21 | .19 |
| Impulsivity/   Behavioral Problems | .23^*^ | .23^*^ | .30^***^ | .32^**^ | .35^***^ | -.05 | .27^*^ | .52^***^ | .32^***^ | .19 | .48^***^ | -.07 | -.11 | .22^*^ | .30^***^ | .19^*^ | -.19 | .16 |
| Inattentiveness/  School Problems | .31^***^ | .31^***^ | .34^***^ | .40^***^ | .21 | -.11 | .36^***^ | .48^***^ | .32^***^ | .09 | .50^***^ | -.03 | -.21 | .15 | .40^***^ | .33^***^ | -.15 | .16 |
| Self-esteem/  Negative Mood | .35^***^ | .38^***^ | .37^***^ | .45^***^ | .23^*^ | -.14 | .39^***^ | .57^***^ | .38^***^ | .02 | .46^***^ | -.08 | -.24^**^ | .27^***^ | .52^***^ | .42^***^ | -.21 | .19 |

**Table S4.** Correlation between Childhood ADHD with its Factor Dimensions and Other Clinical Presentations in **Male Patients with Mood Disorder (*N* = 245)**

^1^The analysis was conducted in the subsamples (n=135) who had the MIQ-T variable.
Note: Age, education, employment, marital status, alcohol use status, and smoking status were adjusted; Adjusted p-values with Bonferroni’s correction were presented ^*^*p*<.05, ^**^*p*<.01, ^***^*p*<.001;
The subdomains of childhood ADHD symptoms were presented by Impulsivity/Behavioral Problems, Inattentiveness/School Problems, and Self-Esteem/Negative Mood.

*Abbreviations* Zung SDS: Zung SDS: Zung Self-Rating Depression Scale; BAI: Beck Anxiety Inventory; TEMPS-A-SV: Temperament Evaluation of Memphis, Pisa, Paris, and San Diego- Autoquestionnaire-short version; MIQ-T: MIQ-T: Mood Instability Questionnaire-Trait; BIS/BAS: The Behavior Inhibition System and Behavior Activation System scales; ASRS: Adult ADHD Self-Report Scale; ERSQ: Emotion Regulation Skills Questionnaire; CD-RISC: Connor–Davidson Resilience Scale ; AUDIT: The Alcohol Use Disorder Identification Test-Korean version; CTQ: Childhood Trauma Questionnaire-short form; IPSM: Interpersonal Sensitivity Measure; CSM: Composite Scale of Morningness; SPAQ: Seasonal Pattern Assessment Questionnaire.

| Variable | **Zung SDS** | **BAI** | TEMPS-A-SV (mood temperament) | | | | | **MIQ-T^1^** | **BIS** | **BAS** | **ASRS** | **ERSQ** | **CD-RISC** | **AUDIT** | **CTQ** | **IPSM** | **CSM** | **SPAQ** |
| --- | --- | --- | --- | --- | --- | --- | --- | --- | --- | --- | --- | --- | --- | --- | --- | --- | --- | --- |
|  |  |  | **Cyclothymic** | **Depressive** | **Irritable** | **Hyperthymic** | **Anxious** |  |  |  |  |  |  |  |  |  |  |  |
| Childhood ADHD:  Total score | .20^***^ | .24^***^ | .28^***^ | .30^***^ | .32^***^ | -.06 | .12 | .52^***^ | .20^***^ | .11 | .41^***^ | -.10 | -.19^**^ | .14 | .53^***^ | .29^***^ | -.15 | .11 |
| Impulsivity/   Behavioral Problems | .13 | .17^**^ | .25^***^ | .25^***^ | .37^***^ | -.01 | .08 | .49^***^ | .12 | .12 | .33^***^ | -.08 | -.14 | .13 | .44^***^ | .20^***^ | -.14 | .10 |
| Inattentiveness/  School Problems | .22^***^ | .24^***^ | .25^***^ | .29^***^ | .22^***^ | -.07 | .15^*^ | .43^***^ | .19^***^ | .08 | .46^***^ | -.10 | -.18^**^ | .12 | .44^***^ | .30^***^ | -.14 | .09 |
| Self-esteem/  Negative Mood | .22^***^ | .26^***^ | .24^***^ | .29^***^ | .19^**^ | -.09 | .13 | .44^***^ | .28^***^ | .06 | .32^***^ | -.10 | -.20^***^ | .13 | .56^***^ | .34^***^ | -.10 | .11 |

**Table S5.** Correlation between Childhood ADHD with its Factor Dimensions and Other Clinical Presentations in **Female Patients with Mood Disorder (*N* = 510)**

^1^The analysis was conducted in the subsamples (n=295) who had the MIQ-T variable.
Note: Age, education, employment, marital status, alcohol use status, and smoking status were adjusted; Adjusted p-values with Bonferroni’s correction were presented ^*^*p*<.05, ^**^*p*<.01, ^***^*p*<.001;
The subdomains of childhood ADHD symptoms were presented by Impulsivity/Behavioral Problems, Inattentiveness/School Problems, and Self-Esteem/Negative Mood.

*Abbreviations* Zung SDS: Zung SDS: Zung Self-Rating Depression Scale; BAI: Beck Anxiety Inventory; TEMPS-A-SV: Temperament Evaluation of Memphis, Pisa, Paris, and San Diego- Autoquestionnaire-short version; MIQ-T: Mood Instability Questionnaire-Trait; BIS/BAS: The Behavior Inhibition System and Behavior Activation System scales; ASRS: Adult ADHD Self-Report Scale; ERSQ: Emotion Regulation Skills Questionnaire; CD-RISC: Connor–Davidson Resilience Scale ; AUDIT: The Alcohol Use Disorder Identification Test-Korean version; CTQ: Childhood Trauma Questionnaire-short form; IPSM: Interpersonal Sensitivity Measure; CSM: Composite Scale of Morningness; SPAQ: Seasonal Pattern Assessment Questionnaire.

**References**

1.	Zung, W.W., *Zung self-rating depression scale and depression status inventory*, in *Assessment of depression*. 1986, Springer. p. 221-231.

2.	Lee, Y.H. and J.Y. Song, *A study of the reliability and the validity of the BDI, SDS, and MMPI-D scales.* Korean Journal of Clinical Psychology, 1991. **10**(1): p. 98-113.

3.	Merikangas, K.R., et al., *Lifetime prevalence of mental disorders in US adolescents: results from the National Comorbidity Survey Replication–Adolescent Supplement (NCS-A).* Journal of the American Academy of Child & Adolescent Psychiatry, 2010. **49**(10): p. 980-989.

4.	Lee, H.-K., et al., *Psychometric properties of the Beck Anxiety Inventory in the community-dwelling sample of Korean adults.* Korean Journal of Clinical Psychology, 2016. **35**(4): p. 822-830.

5.	Choi, S., et al., *Korean validation of the short version of the TEMPS-A (Temperament Evaluation of Memphis, Pisa, Paris, and San Diego Autoquestionnaire) in patients with mood disorders.* Medicina, 2023. **59**(1): p. 115.

6.	Akiskal, H.S., et al., *TEMPS-A: progress towards validation of a self-rated clinical version of the Temperament Evaluation of the Memphis, Pisa, Paris, and San Diego Autoquestionnaire.* Journal of affective disorders, 2005. **85**(1-2): p. 3-16.

7.	Matsumoto, S., et al., *Reliability and validity of TEMPS-A in a Japanese non-clinical population: application to unipolar and bipolar depressives.* Journal of affective disorders, 2005. **85**(1-2): p. 85-92.

8.	Yoon, J., et al., *Development and Validation of the Mood Instability Questionnaire-Trait (MIQ-T).* Medicina, 2021. **57**(8): p. 838.

9.	Carver, C.S. and T.L. White, *Behavioral inhibition, behavioral activation, and affective responses to impending reward and punishment: the BIS/BAS scales.* Journal of personality and social psychology, 1994. **67**(2): p. 319.

10.	Kim, K.-h. and W.-s. Kim, *Korean-BAS/bis scale.* Korean Journal of Health Psychology, 2001. **6**(2): p. 19-37.

11.	Kim, J.-H., E.-H. Lee, and Y.-S. Joung, *The WHO Adult ADHD Self-Report Scale: reliability and validity of the Korean version.* Psychiatry investigation, 2013. **10**(1): p. 41.

12.	Berking, M. and H. Znoj, *Entwicklung und Validierung eines Fragebogens zur standardisierten Selbsteinschätzung emotionaler Kompetenzen (SEK-27).* Zeitschrift für Psychiatrie, Psychologie und Psychotherapie, 2008. **56**(2): p. 141-153.

13.	Berking, M. and H. Znoj, *SEK-27-Fragebogen zur standardisierten Selbsteinschätzung emotionaler Kompetenzen.* 2011.

14.	Do, H.S., et al., *Relationship between emotion regulation skills, resilience, depression and anxiety symptom severity in patients with mood disorders and non-clinical participants: a mediation model.* European Archives of Psychiatry and Clinical Neuroscience, 2025: p. 1-15.

15.	Connor, K.M. and J.R. Davidson, *Development of a new resilience scale: The Connor‐Davidson resilience scale (CD‐RISC).* Depression and anxiety, 2003. **18**(2): p. 76-82.

16.	Jung, Y.E., et al., *The Korean version of the Connor–Davidson Resilience Scale: an extended validation.* Stress and Health, 2012. **28**(4): p. 319-326.

17.	Choi, S., et al., *The moderating effect of resilience on the relationship between academic stress and school adjustment in Korean students.* Frontiers in psychology, 2023. **13**: p. 941129.

18.	Lee, K.-U., et al., *Psychometric properties of the Korean version of the Interpersonal Sensitivity Measure (IPSM-K).* Comprehensive psychiatry, 2013. **54**(7): p. 918-924.

19.	Boyce, P. and G. Parker, *Development of a scale to measure interpersonal sensitivity.* Australian and New Zealand journal of psychiatry, 1989. **23**(3): p. 341-351.

20.	Bernstein, D.P., et al., *Development and validation of a brief screening version of the Childhood Trauma Questionnaire.* Child abuse & neglect, 2003. **27**(2): p. 169-190.

21.	Lee, B., *Development of Korean version of alcohol use disorders identification test (AUDIT‐K): Its reliability and validity.* Journal of Korean Academy of Addiction Psychiatry, 2000. **4**: p. 83.

22.	Kim, C.G., et al., *Reliability and validity of alcohol use disorder identification test-Korean revised version for screening at-risk drinking and alcohol use disorders.* Korean J Fam Med, 2014. **35**(1): p. 2-10.

23.	Smith, C.S., C. Reilly, and K. Midkiff, *Evaluation of three circadian rhythm questionnaires with suggestions for an improved measure of morningness.* Journal of Applied psychology, 1989. **74**(5): p. 728.

24.	Kim, J.K., *Circadian rhythm and psychological adjustment.* The Korean Journal of Health Psychology, 1998. **3**(1): p. 129-140.

25.	Magnusson, A., *Validation of the seasonal pattern assessment questionnaire (SPAQ).* Journal of affective disorders, 1996. **40**(3): p. 121-129.

26.	Kim, S.-J., et al., *Seasonal variations of mood and behavior in Korean medical students.* Sleep Medicine and Psychophysiology, 2004: p. 95-99.
